# A resource-based game theoretical approach for the paradox of the plankton

**DOI:** 10.7717/peerj.2329

**Published:** 2016-08-18

**Authors:** Weini Huang, Paulo Roberto de Araujo Campos, Viviane Moraes de Oliveira, Fernando Fagundes Ferrreira

**Affiliations:** 1 Department Evolutionary Theory, Max Planck Institute for Evolutionary Biology, Plön, Germany; 2 Centre for Tumour Biology, Barts Cancer Institute, Queen Mary University of London, London, United Kingdom; 3 Evolutionary Dynamics Lab, Department of Physics, Federal University of Pernambuco, Recife, PE, Brazil; 4 Department of Physics, Federal Rural University of Pernambuco, Recife, PE, Brazil; 5 Center for Interdisciplinary Research in Complex Systems, Universidade de São Paulo, São Paulo, SP, Brazil

**Keywords:** Evolutionary game theory, Species interactions, Biodiversity, Plankton paradox, Resource-based model

## Abstract

The maintenance of species diversity is a central focus in ecology. It is not rare to observe more species than the number of limiting resources, especially in plankton communities. However, such high species diversity is hard to achieve in theory under the competitive exclusion principles, known as the plankton paradox. Previous studies often focus on the coexistence of predefined species and ignore the fact that species can evolve. We model multi-resource competitions using evolutionary games, where the number of species fluctuates under extinction and the appearance of new species. The interspecific and intraspecific competitions are captured by a dynamical payoff matrix, which has a size of the number of species. The competition strength (payoff entries) is obtained from comparing the capability of species in consuming resources, which can change over time. This allows for the robust coexistence of a large number of species, providing a possible solution to the plankton paradox.

## Introduction

Species coexistence under ecological competition has been long debated in ecology (May & Angela, 2007). A fundamental question is how it is possible to observe so many species coexisting as few limiting resources are available. This pattern is not rare in natural communities, being the best example the observed species diversity of algae communities with two or three limiting resources in relatively homogenous environment. This contradicts the predictions made by several theoretical models which conclude that no more species can coexist compared to the number of limiting resources in a homogenous environment (Petersen, 1975; Grover, 1997). This inconsistency between theoretical results and observations is named the paradox of plankton (Hutchinson, 1961).

One of the first theoretical models on species competition is the competitive Lotka–Volterra model (Lotka, 1925; Volterra, 1926). Originally, the model describes two-species interactions, where the growth of one species linearly decreases with the densities of itself and its competitor. The two species stably coexist with each other when the intraspecific competition is stronger than the interspecific competition. It was later generalised to take into account competition among three or more species (May & Leonard, 1975; Hofbauer & Sigmund, 1998). Although more complex dynamics, e.g., limit cycles of species, can arise compared to the two-species model, it is also shown that the difficulty of attaining a stable coexistence of all species increases with the number of species (Strobeck, 1973; Gokhale & Traulsen, 2010). In the Lotka–Volterra model, interspecific differences are required among coexisting species. Identical or equivalent species refer to the same growth rates, carrying capacities and competition coefficients. The competition dynamics of identical species is under a neutral process, and random drift will lead to the extinction of one species if the population size is finite.

The Lotka–Volterra model indicates the change of density under pairwise competition and does not include how species use the resources. Instead, the resource-based competition theory is more relevant in the study of species coexistence under limiting resources. Species interact through their consumption on common resources. The growth rate of a species is coupled to the availability of resources, which is related to both the resource amount and the number of consumers (Petersen, 1975; Tilman, 1976; Tilman, 1982). In this framework, many species may use the same resource, and therefore it is not constrained to pairwise species competitions.

In resource-based competition theory, a commonly used form for the specific growth rate is the Michaelis-Menton or Monod equations (Monod, 1950; Johnson & Goody, 2011). Formerly it had often been employed to describe the growth of microorganisms like marine phytoplanktons (Dugdale & Goering, 1967). The growth rate of a species *i* by using a specific resource *m* is defined as ${r}_{i} \frac{{R}_{m}}{{R}_{m}+{K}_{m,i}} $. Here, *r*_*i*_ is the maximal specific growth rate of this species, *R*_*m*_ the available amount of the resource, and *K*_*m*,*i*_ is the half-saturation constant of species *i* to resource *m*, the resource level when the species grows at a rate of $ \frac{{r}_{i}}{2} $. While *R*_*m*_ is a quantity that changes with the resource supply and the consumption of the species using the resource. The quantities *r*_*i*_ and *K*_*m*,*i*_ are often considered as experimental coefficients.

Resource-based competition models have been used to predict the results of species competition in experiments (Hansen & Hubbel, 1980; Sommer, 1986; Wedin & Tilman, 1993). These models propose a simple *R*^∗^-rule for species competition, where *R*^∗^ means the minimum amount of a resource required to sustain a species. It states that the dominant competitor is the one with the lowest *R*^∗^ when species compete for one common resource. This confirms the competitive exclusion principle (Hardin, 1960), and leads to the conclusion that the maximum number of species that can be maintained in a community is the same as the number of distinct resources (Petersen, 1975). Although competition exclusion is obeyed in a great variety of homogeneous well-mixed environments and corroborated by experimental observations (Gause, 1932; Gause, 1934; Park, 1948), this tenet is clearly violated in many other cases and most natural communities are rich in species (Hutchinson, 1961; Clark et al., 2007).

Heterogeneous or temporarily changing environment may lead to higher species diversity than predicted by the competition exclusion principle (Tilman, Mattson & Langer, 1981; Powell & Richerson, 1985; Holt, 2001; Descamps-Julien & Gonzalez, 2005). Another possibility is that ecological systems can be driven by biotic feedback instabilities that keep the competing population out of equilibrium (DeAngelis & Waterhouse, 1987). These internal system feedbacks can generate strong oscillations, which sustain a large number of species coexisting than allowed by the competitive exclusion principle (Armstrong & McGehee, 1976; Huisman & Weissing, 1999; Huisman & Weissing, 2002; Revilla & Weissing, 2008). These oscillations often happen in certain conditions, where specific species are required. It is claimed that these conditions refer to a restrictive set of the parameter space and are vulnerable to small parameter changes or mutations (Schippers et al., 2001; Shoresh, Hegreness & Kishony, 2008). Recent research shows that in addition to physical forcing such as continual temporal and spatial changes of the ocean surface boundary layer, phytoplankton variability can be ascribed to interspecies competition (Kenitz et al., 2013). Large contrasts in half-saturation coefficients can promote oscillatory and chaotic dynamics, which sustains a large number of species. A high level of phytoplankton diversity can be achieved at low nutrient environments both in experiments (Leibold, 1999) and theoretical studies (Tubay et al., 2013). Other possible mechanisms of the high species richness in nature include emergent neutrality where species have similar fitness within clumps (Segura et al., 2011), simultaneous multiple resource limitation which leads to complex dynamics including non-equilibrium states as oscillations and chaos (Dutta, Kooi & Feudel, 2014) and production of toxin by some species (Chakraborty, Ramesh & Dutta, 2016; Fernández et al., 2016).

Despite these recent advances, the maintenance of high species diversity under limiting resources especially in a homogeneous constant environment is still unclear in a general perspective. Here we propose a resource-based model of multi-species competition in the framework of evolutionary game theory, which allows for stable coexistence of a large number of species in a relatively large parameter range. Species compete for common resources and space. The interactions among species are frequency dependent, which means that the growth of one species does not only depend on itself but also on the frequencies of other species in the community. Frequency dependent interactions abound in distinct populations such as plants (Falster & Westoby, 2003), yeast (MacLean & Gudelj, 2006; MacLean et al., 2010) and bacteria (Levin, Antonovics & Sharma, 1988). Evolutionary game theory is a methodology to capture frequency dependent interactions among different species. The equivalence between evolutionary game theory and the classical Lotka–Volterra model has already been reported (Hofbauer & Sigmund, 1998). By integrating two different approaches, i.e., evolutionary game theory and resource-based competition theory, we are interested to understand whether complex individual interactions and limiting factors will result in higher species diversity in a constant environment.

While species do coexist in ecological time scales, natural communities are also often under non-equilibrium conditions and subjected to the appearance of new species and extinction (Hubbell, 2001). Thus, we address the question from a combined ecological and evolutionary perspective. Instead of assuming predefined species (Huisman & Weissing, 1999; Huisman & Weissing, 2001; León & Tumpson, 1975; Tilman, 1982), we consider an evolutionary process where spontaneous mutations arise and bring variety in the community. We see that selection will lead to the stable coexistence of different species in a ecological time scale before new mutations become prevalent, which differs from a temporary diversity only arising from random mutations and drift. Most mutations will be removed under selection and drift, but some will remain and finally break the coexistence of previous resident species and lead to a new equilibrium of the community. Our framework is in line with evolutionary experiments which unveil the evolutionary responses of phytoplankton communities to environmental changes such as ocean acidification (Collins, Rost & Rynearson, 2014; Collins, 2011). Evidence of the potential genetic variation in these communities are found in isolates of species sampled from distinct locations (Rynearson & Virginia Armbrust, 2004; Weynberg et al., 2011), especially under stressful conditions such as temperature increase (Huertas et al., 2011) and contamination (Huertas et al., 2010). The relevant mutation rates in such populations lie within a range of 10^−5^–10^−7^ (López-Rodas et al., 2007; López-Rodas et al., 2008).

Competition at the individual level are settled by comparing their requirements and capabilities in managing the available resources. Competition at the species level is described as a dynamical payoff matrix between different species. The number of species in the payoff matrix is not prefixed, but changes due to mutation and extinction. Species go extinct under selection and drift and new species arise from extant species. The capability of the species to extract energy for growth evolves through mutations. A mutation is referred to as the appearance of a new species, which differs from the standard interpretation of mutations as genetic alteration in organisms. Every new species has different characteristics in handling the resources compared with the extant species. As a consequence, the emerging biodiversity is the result of an evolving process.

## Materials and Methods

We consider a community composed of different species in the same trophic level. The number of species has a dynamical value, which can decrease due to extinction or increase because of the appearance of new species. Every species has a certain number of individuals, which compete for common resources. The reproduction is under the interplay of selection and random drift. Individuals reproduce randomly but proportionally to their fitness. Thus, the abundance of every species may change over time. There is limited space in the community, which is implemented by considering a fixed community size *N*. We consider a closed system, i.e., there is no immigration from outside of the community or meta-population structures. Individuals from the same species have the same fitness being determined by the species’ capability to use the resources and by the interactions with other individuals in the community.

Our model mimics a community composed of multiple resources and multiple species. The capability of a given species in using a given resource is defined by a Monod-like function (see below). Based on the calculated values of species’ capabilities in using resources, we define the payoff matrix capturing the interspecific and intraspecific competitions. The fitness of a species—which is the average number of offspring of an individual in the species—depends on itself and its competitions with other species in the same community, thus on the payoff matrix and all species frequencies.

### Competition on resources

We first look at the capability of different species in making use of the resource. *M* is the total number of resource types available for the community. Each species consumes only a subset of *M*_cons_ ≤ *M* of the resources. The subset used by each species is randomly chosen among the *M* resources and does not change over time for the corresponding species. Note that *M*_cons_ = *M* means that there is a complete overlap among the resources of all species, and *M*_cons_ < *M* corresponds to a more realistic assumption whereby species are differentiated from each other (Connell, 1980; Schoener, 1974).

We assume a Monod-like function to describe the capability of species *i* in using its resource *m*, $ \frac{{R}_{m}}{{R}_{m}+{K}_{m,i}} $. *R*_*m*_ denotes the amount of resource *m* available for one individual, i.e., ${R}_{m}={R}_{m}^{{^{\prime}}}/{N}_{m}$, and *N*_*m*_ is the number of individuals consuming resource *m* at the current time in the whole community. Here ${R}_{m}^{{^{\prime}}}$ is drawn from an uniform distribution (0, *R*_Max_), and *R*_Max_ is the maximum value for any resource type, a parameter of the model. The half-saturation constant *K*_*m*,*i*_ means how efficiently species *i* synthesizes resource *m* into its internal energy. Each species has a set of half-saturation constants {*K*_*m*,*i*_} with size *M*_cons_. These are random variables fulfilling the condition, ∑_*m*_*K*_*m*,*i*_ = *M*_cons_∕2. This condition assures that a given species cannot achieve high efficiency in transforming all the resources into its internal energy. Otherwise, the ultimate fate of the evolutionary process would be the fixation of a single species that can survive by consuming nearly no resources. This trade-off in the utilization of resources is reported to be a necessary condition for coexistence (Vincent et al., 1996).

All the *M*_cons_ resources used by a given species are assumed to be essential, and hence the success of a given species is determined by the resource that is the most limiting. This assumption corresponds to the Von Liebig’s “Law of the Minimum”. The interaction of species *i* with the resources is quantified by the function

(1) \begin{eqnarray*}{\mu }_{i}=\min \left\{ \frac{{R}_{1}}{{R}_{1}+{K}_{1,i}} , \frac{{R}_{2}}{{R}_{2}+{K}_{2,i}} ,\ldots , \frac{{R}_{{M}_{\mathrm{cons}}}}{{R}_{{M}_{\mathrm{cons}}}+{K}_{{M}_{\mathrm{cons}},i}} \right\} ,\end{eqnarray*}

where min refers to the minimum function. The form of *μ*_*i*_ is similar as the growth rate of species in the framework of infinite populations where dynamics is described by a set of differential equations (Huisman & Weissing, 1999).

### Payoff matrix

Next we consider the interspecific and intraspecific competition between individuals. Individuals compete due to the limited capacity of the system. Those with a higher capability in exploiting the resources have an advantage in such a competition. At each time step every individual interacts with the *N* − 1 remaining individuals in the community. The payoff entries of these interactions are given by

(2) \begin{eqnarray*}{a}_{ij}= \left\{ \begin{array}{@{}ll@{}} \displaystyle \frac{-1}{1+\exp \nolimits ({\mu }_{i}-{\mu }_{j})} &\displaystyle \text{if}~i\not = j,\\ \displaystyle {\mu }_{i}-1&\displaystyle \text{if}~i=j, \end{array} \right. \end{eqnarray*}

which lies in the range (−1, 0). The element *a*_*ij*_ with *i* ≠ *j* quantifies the average gain of an individual of species *i* in an interspecific competition, i.e., it competes with an individual of species *j*. The element *a*_*ii*_ describes its gain in an intraspecific competition. The smaller the payoff entry is, the smaller the gain in the corresponding competition. Note that the matrix is not symmetrical. For any *i* ≠ *j*, if *μ*_*i*_ < *μ*_*j*_, *a*_*ij*_ is closer to −1 than *a*_*ji*_. The competitive interaction is more hostile for species *i*, as species *j* can make more efficient use of the resources than species *i*, *μ*_*i*_ < *μ*_*j*_. When *i* = *j* and *μ*_*i*_ approaches one, the element *a*_*ii*_ becomes close to zero and the intraspecific competition is mitigated because as this situation corresponds that resources are plentiful. An important feature in the definition of the payoff matrix is that it provides a mechanistic approach for the competition model since the intraspecies and interspecific interactions are both dependent on species’ properties only.

### From payoffs to fitness

In a well-mixed population, the average payoff of an individual from species *i* is estimated as

(3) \begin{eqnarray*}{\pi }_{i}= \frac{1}{N-1} \left[ \sum _{j=1}^{n}{a}_{ij}{N}_{j}-{a}_{ii} \right] ,\end{eqnarray*}

where *n* denotes the number of species varying over time, and *N*_*j*_ is the number of individuals of species *j* and ${\mathop{\sum }\nolimits }_{j=1}^{n}{N}_{j}=N$. Now we map the payoff to fitness, which determines the number of offspring that an individual has. The average fitness of an individual of species *i* is defined as the exponential of its payoff, *f*_*i*_ = *e*^+*w*⋅*π*_*i*_^, and *w* plays the role of a selection intensity (0 < *w* < ∞) (Traulsen, Shoresh & Nowak, 2008). The exponential fitness mapping function can avoid negative fitness values for any payoff value.

### Mutations in a Wright–Fisher process

We set the community isogenic in the beginning and let it evolve under mutations. The reproduction and death of individuals follow a Wright–Fisher process. In every time step, all individuals randomly produce a certain number of offspring proportional to their fitness. The individuals whose fitness is larger than the average fitness of all individuals in the community will produce more offspring than the others. From the offspring pool, *N* individuals are randomly sampled to form the community in the next time step. A Wright–Fisher process represents non-overlapping generations. However, it often quantitively leads to the same dynamic pattern as other models with overlapping generations especially when selection is weak, e.g., a Moran process where only one individual reproduces and one individual dies in every time step.

During reproduction, an individual produces offspring as itself with the probability 1 − *ν* and gives rise to a new species with the probability *ν*, i.e., speciation is analogous to point mutation (Hubbell, 2001; Chave, 2004). Each newly arisen species entails a new subset of *M*_cons_ resource types and their corresponding half-saturation constants, *K*-values. The way of describing mutations and extinctions are inspired by the method of Huang et al. in modelling random mutants (Huang et al., 2012). However, the present model differs from that framework in two key aspects: the payoff entries in this model are obtained based on the capability of species in handling the resources; the payoff entries are evaluated every generation as the number of individuals in different species can change in every time step.

## Results

**Figure 1:**
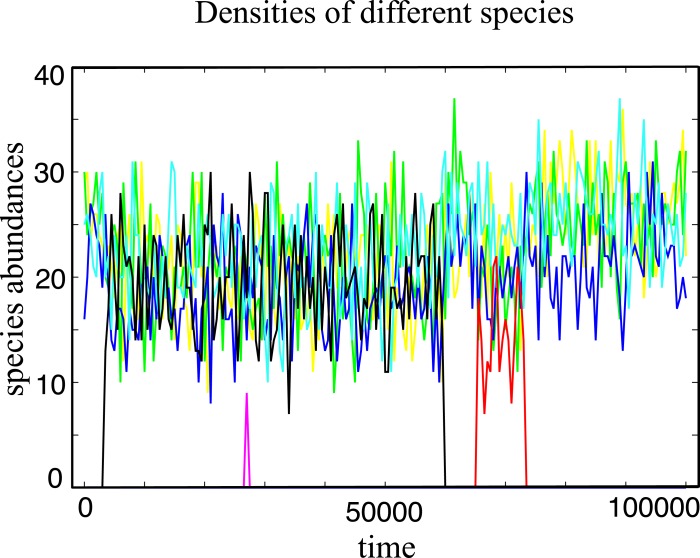
Typical evolutionary trajectory of a single community. The community starts with only one species, and after it evolves in 10^7^ generations we record the number of individuals for every species in the next 10^5^ generations. In this time window, we have around 4 spontaneous mutations in total. Some of them go extinct in a short time and some of them (e.g., the black line and the red line) coexist with other species for a long time period under strong selection. The Parameter sets: *N* = 100, *w* = 10, *M* = 3, *M*_cons_ = 2, *R*_Max_ = 30 and *ν* = 5 × 10^−7^.

A typical evolutionary trajectory in a community of *N* = 100 individuals under strong selection is shown in Fig. 1. The coexistence of a considerable quantity of distinct species is observed, promoting a high species diversity level in the community. In the model, species have a finite lifespan being replaced by a better competitor or by ecological drift (Hubbell, 2001), a mechanism not present in infinitely large populations.

We also measure the average species diversity after a community has evolved to a steady regime from the initial state. Under neutral selection, the expected number of species is calculated in the unified neutral theory of biodiversity and biogeography as

**Figure 2:**
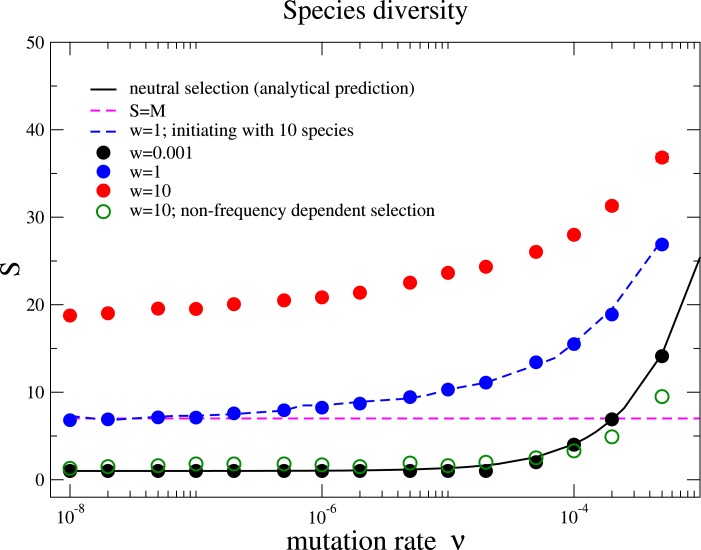
Species diversity *S* versus mutation rate *ν* under different selection intensities. The solid line is the theoretical prediction of the neutral theory given by Eq. (4) in the main text, which fits well with our simulation results under near neutral selection *w* = 0.001. The species diversity increases when the selection intensity becomes stronger (*w* = 1 and *w* = 10). This holds under different initial conditions with a homogenous community or a community with multiple species (*w* = 1, blue dots for 1 species and blue dashed line for10 species with random half-saturation constants). In a large parameter range, the average number of species can be above the total number of limiting resources in the community (the pinked dashed line for *S* = *M*). In addition, the species diversity is much lower if interactions between different species are frequency independent (comparing the green unfilled points with the red points under *w* = 10). The Parameter sets: *N* = 2, 000, *M* = 7, *M*_cons_ = 5, and *R*_Max_ = 300. (The results are averaged over 10^6^ generations after the community has evolved for 10^8^ generations.)

(4) \begin{eqnarray*}S= \frac{\theta }{\theta } + \frac{\theta }{\theta +1} + \frac{\theta }{\theta +2} +\cdots + \frac{\theta }{\theta +N-1} ,\end{eqnarray*}

where *θ* = 2*Nν* is referred to the fundamental biodiversity number under the Wright–Fisher process (Hubbell, 2001). For weak selection, *w* = 0.001, the species diversity coincides with the above prediction under various mutation rates, Fig. 2. This is because the unified neutral theory of biodiversity and biogeography presumes complete ecological equivalence at the species and the individual levels, and thus refers to the case of the nearly neutral selection in our model. We observe that the diversity *S* increases with the mutation rate *ν* and exceeds the total number of resources for some mutation rates for *w* = 0.001. However, the diversity is driven by the recurrent mutations instead of selection.

Now we move beyond the main premise of the neutral theory, and discuss the situation whereby species are not ecologically equivalent but differ in their capabilities of using resources. This refers to larger selection intensities in the model, and now the negative frequency-dependent selection plays a prominent role in fostering diversity. Negative frequency-dependent selection means that intraspecific competition is stronger than the interspecific competition, a required condition for stable coexistence among species (Chesson, 2000). Under negative frequency-dependent selection, a species enjoys an advantage compared to its opponent species while rare, but loses this advantage when it becomes abundant. Our results show that the species diversity increases with the selection intensity, see Fig. 2. This is opposed to the expected outcome in communities under constant selective pressure, where the fitness of each species is constant and does not depend on the composition of the whole community.

It is important to highlight that the coexistence among the different species is obtained not by specifying a prior the number of species as in previous formulations or by a predefined payoff matrix. Instead, it is an emerging property under the balance between natural selection, drift and speciation. While the emergence of a stable diversity is also observed in a random mutant model without species-resource level interactions, we observe considerably higher diversity in our model (Huang et al., 2012). Even though the mutation rate is relatively low, the species diversity can be larger than the number of resources available for the community.

**Figure 3:**
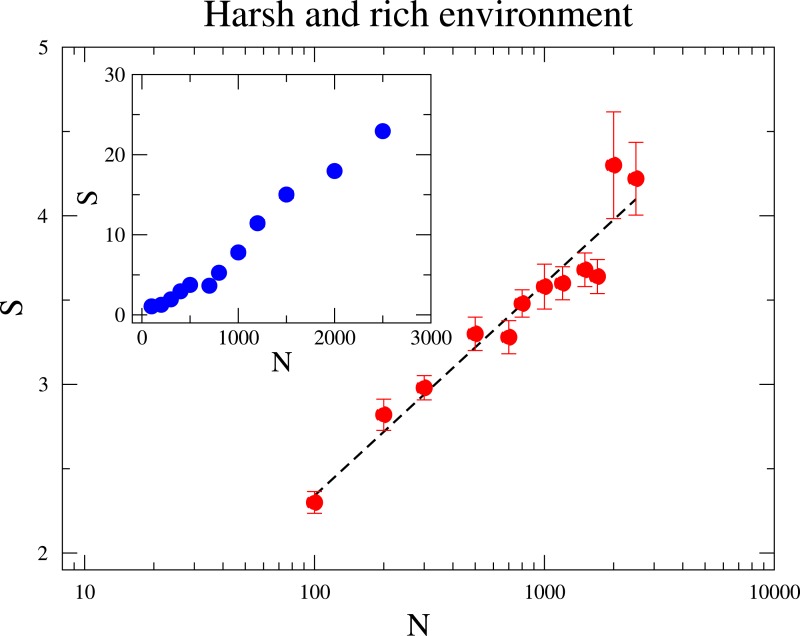
The dependence of the diversity *S* with the community size *N*. In the main figure, *R*_Max_∕*N* is fixed to be 1∕2, i.e., *R*_Max_ is varied as the community size *N* changes. In the inset *R*_Max_ is constant and equal to 300. The parameter values are *M* = 7, *M*_cons_ = 2 and mutation rate *ν* = 1 × 10^−6^. (The results are averaged over 10^6^ generations after the community has evolved for 10^8^ generations.)

At the sequel we address the relationship between the species diversity and the community size. In Fig. 3, we investigate two distinct scenarios: in the inner panel, the maximum amount of resource *R*_Max_ is constant under varying community sizes; In the outer panel, the ratio *R*_Max_∕*N* is fixed such that an increased community size *N* does not mean a more harsh environment. In both scenarios diversity grows with *N*, but the relationships that best describe the dependence of diversity on *N* are quite distinct. When *R*_Max_ is constant, diversity rises linearly with *N*. This means that scarcity of resources favours the coexistence of more species, which agrees with pervious research (Tubay et al., 2013). This is because a species is less likely to reach higher frequencies under large *N*, as the environment becomes more hostile to a given species and the intraspecific competition is enhanced. On the other hand, when *R*_Max_∕*N* is kept fixed for various *N*, the increase of diversity with *N* is logarithmic, as it can be likewise observed in neutral communities (Hubbell, 2001). This observation seems to evince that under the second scenario the augment of *S* with *N* owes mostly to the reduction of the strength of ecological drift.

**Figure 4:**
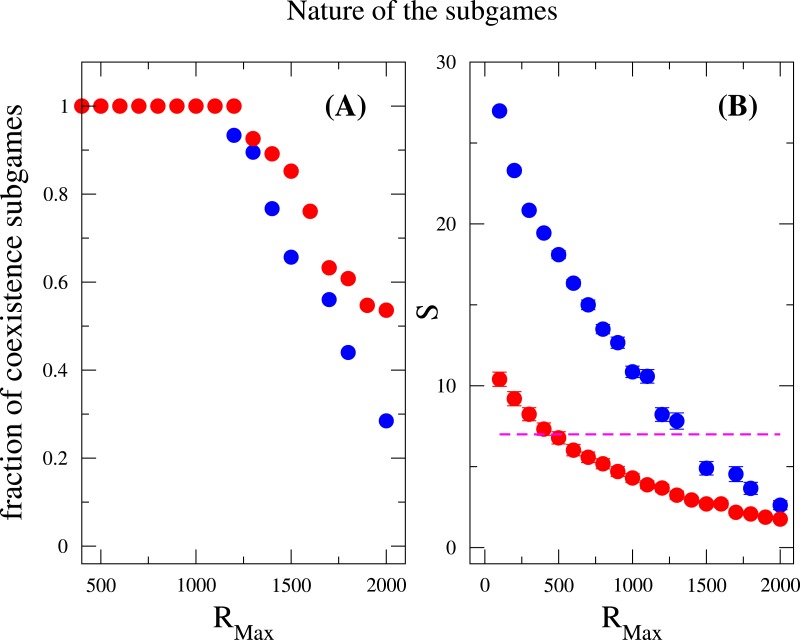
Nature of the subgames between any two species in a community. Fraction of coexistence games (A) and species diversity *S* (B) are plotted as a function of *R*_Max_. Blue circles are results for *w* = 10, and red circles are for *w* = 1. The dashed line (in B) indicates that the average number of species is the same as the total number of resources available, i.e., *S* = *M* = 7. The fraction of coexistence game is close to one when *R*_Max_ < 1, 200, and decreases when *R*_Max_ increases further. On the other hand, the species diversity drops off for large values of *R*_Max_. This can be related to both the increase of *R*_Max_∕*N* ratio and the decrease of the fraction of coexistence games. The parameter sets: *N* = 2, 000, *M* = 7, *M*_cons_ = 5, *ν* = 1 × 10^−6^. (The results are averaged over 10^6^ generations after the community has evolved for 10^8^ generations.)

Last but not least, we discuss how does the nature of the subgames between any two species contributes to the species diversity. Suppose the payoff entries of two species are *a*_11_, *a*_12_, *a*_21_ and *a*_22_. We examined the fraction of coexistence games characterized by the conditions *a*_21_ > *a*_11_ and *a*_12_ > *a*_22_, prisoner-dilemma (*a*_21_ > *a*_11_ > *a*_22_ > *a*_21_) and bistability (or coordination games, *a*_11_ > *a*_21_ and *a*_22_ > *a*_12_) under strong selection. Again, these measurements were done after the community has attained the equilibrium state. As previously mentioned, the coexistence game is prevalent among all subgames for a broad range of *R*_Max_ (*R*_Max_ < 1, 200), where neither coordination games nor Prisoners’ dilemma games are observed, see Fig. 4. However, when *R*_Max_ ≃ 1,200, an abrupt soaring of the fraction of coordination games is verified and an abrupt drop of coexistence games ensues. In addition, the species diversity *S* decreases when *R*_Max_ increases, which is consistent with the result in the inset of Fig. 3. For a given *N*, increasing *R*_Max_ means increasing the maximum possible resources for every individuals. From these, we conclude that coexistence game promotes high species diversity, and coordination games are very unlikely to appear in a scarcity of resources, i.e., *R*_Max_ is not large enough. A similar outcome has been found concerning behavior polymorphism in fruitfly populations Fitzpatrick et al. (2007), where low nutrient conditions enhanced behavior polymorphism. Negative-frequency dependent selection has also been shown to play a role in the maintenance of diversity in clonal populations of mite Weeks & Hoffmann (2008). It has argued that negative frequency-dependent interactions spontaneously arise as a result of differential resource utilization even in clonal populations.

## Discussion

The study of species competition has focused on mechanisms that can underpin high biodiversity observed in nature. According to the competitive exclusion principle, species diversity at equilibrium is limited by the number of limiting resources. In the work presented here, we model frequency dependent competitions among species for common resources and limited capacity of the system. Species can evolve during the process. A large number of species can coexist under the balance of intraspecific and interspecific interactions.

The complex interactions among evolving species can be qualitatively depicted through the game theory formalism. We define the payoff matrix as a function of specific growth rates, which depends on the resource availability. Note that we define that the growth of a species is limited by the resource with the lowest availability or utility, however, more complicated scenarios where growth is limited by several resources simultaneously do exist (Harpole et al., 2011). This co-limitation of growth can be a future direction of extensions for our model. During the interaction, species population fluctuates in size. Moreover, we allow the half saturation constant to evolve in time, which introduce novelty in the species diversity. However, the reproduction of individuals is subject to natural selection and random drift. Thus, the maintenance of species diversity is under the interplay of mutation, selection and drift.

Among the results shown here, we highlight a robust coexistence of a large number of species, exceeding the total number of limiting resources available to the community. We emphasize that the observed coexistence is not based on chaotic oscillations but obtained as a trade-off between resource requirement selection and adaptation. In addition, when maximum resource supply is constant, diversity rises linearly with the density *N*. This result suggests that intraspecific competition is enhanced by resource scarcity. On the other hand, when *R*_Max_∕*N* is kept fixed for various *N*, the increase of diversity with *N* is logarithmic, and augment of *S* with *N* owes mostly to the reduction of the strength of random drift.

This model yields a dynamical and adaptive aspect for species competition, and enables us to address the emergence and maintenance of species diversity from first principles. For a broad range of parameter sets, the species diversity is considerably high and surpasses the number of resources available to the community, which differs from the standard conclusion that follows from the competitive exclusion principle. In addition, this model brings a connection between evolutionary game theory and resource competition theory, which may shed new light into the investigation of the diversity of species in the context of resource competition.

##  Supplemental Information

10.7717/peerj.2329/supp-1 Supplemental Information 1 C + + Code used to generate the frequencies of the different types over time Code used to produce Fig. 1.

10.7717/peerj.2329/supp-2 Supplemental Information 2 C + + Code used to calculate the average quantities in the equilibrium Code used to produce Figs. 2–4.

## Additional Information and Declarations

### Data Availability

The following information was supplied regarding data availability: The code used to produced all the results and figures of our manuscript are provided as Supplementary Files.
